# NETTAB 2014: From high-throughput structural bioinformatics to integrative systems biology

**DOI:** 10.1186/s12859-016-0907-y

**Published:** 2016-03-02

**Authors:** Paolo Romano, Francesca Cordero

**Affiliations:** Bioinformatics, IRCCS AOU San Martino – IST, c/o Centro Biotecnologie Avanzate (CBA), Largo Rosanna Benzi 10, I-16132 Genoa, Italy; Department of Computer Science, University of Torino, Torino, I-10149 Italy

## Abstract

The fourteenth NETTAB workshop, NETTAB 2014, was devoted to a range of disciplines going from structural bioinformatics, to proteomics and to integrative systems biology. The topics of the workshop were centred around bioinformatics methods, tools, applications, and perspectives for models, standards and management of high-throughput biological data, structural bioinformatics, functional proteomics, mass spectrometry, drug discovery, and systems biology.

43 scientific contributions were presented at NETTAB 2014, including keynote, special guest and tutorial talks, oral communications, and posters. Full papers from some of the best contributions presented at the workshop were later submitted to a special Call for this Supplement.

Here, we provide an overview of the workshop and introduce manuscripts that have been accepted for publication in this Supplement.

## NETTAB Workshops

Network Tools and Applications in Biology (NETTAB) Workshops are a series of International meetings held annually in Italy. The goal of this series of workshops is the study and analysis of the impact that innovative Information and Communication Technologies (ICTs) may have on bioinformatics and biomedical research [1].

During the meeting, scientific sessions, which are usually introduced by keynote talks, are focused on tools, systems and applications that can be conceived and developed by adopting such technologies. Also important is the assessment of their expected impact in bioinformatics and research and discussion among participants within sessions is a key factor for depicting the foreseen impact. A poster session allows all participants to present and discuss their ideas on the main topics, even when they are not already completely developed in a tool or proven useful for research. Usually, the agenda of NETTAB workshops also includes some tutorials on targeted technologies so that participants may also extend their knowledge and awareness on them.

Targeted technology changes every year in order to cope with the continuously evolving technological innovation. Various topics have therefore been focused and discussed, some of which have been very timely, including, among others, XML-based data integration in 2001, scientific workflows in 2005, Web Services in 2006 [2], the Semantic Web in 2007 [3], biological Wikis in 2010, and mobile applications (apps) for Life Sciences in 2013 [4]. A full list of topics and access to web sites is available on the web [1].

## NETTAB 2014: the fourteenth edition

The fourteenth NETTAB workshop, NETTAB 2014, was held on October 15–17, 2014, in Torino, Italy. Workshop chairs were Francesca Cordero, University of Torino, Torsten Schwede, University of Basel, Basel, Switzerland, and Paolo Romano, Cancer Comprehensive Centre and University Hospital San Martino IST, Genoa, Italy.

It was a joint event with the “2014: Crystal (cl)Year” conference, held in the context of the celebrations for the UNESCO International Year of Crystallography 2014, and the annual meeting of the Centre for Complex Systems in Molecular Biology and Medicine of the University of Torino. The workshop aimed at representing a virtual bridge between these two events, showing how to manage and elaborate structural and high-throughput proteomics data so that it may be integrated with information from other life sciences disciplines with the aim of reaching a richer description and a deeper understanding of mechanisms and interactions in the involved system: the human being and its physiological and pathological states.

One of the main themes of the workshop was related to high-throughput proteomic data and their usage in the systems biology. While high-throughput genomic data were from the beginning more available and manageable to systems analysis then proteomic data, recent technological and methodological advances in the latter have contributed to an increase in proteomic studies in a high-throughput way. Combined proteomic technologies can indeed produce complex, integrated datasets that may also constitute a sound support for systems biology research.

Moreover, the usage of proteomic data for structural bioinformatics and integrative systems biology is not yet sufficiently explored. So, the workshop Chairs thought that it was the time to investigate the possible contribution of new ICT technologies to structural bioinformatics, a scientific discipline where computing needs are relevant, especially when evaluated in the light of high-throughput technologies, of the possibilities of analysing human variation both at genomic and at proteomics levels, and of the need of automating all related data analysis procedures.

At the same time, recent advances in genomics and proteomics have produced large datasets enabling for a global analysis of molecular interactions. The integration of computational models and experimental results that is made possible by Systems Biology allows to describe and understand the dynamic properties of biological systems, which are seen as complex signalling networks.

The topics of the workshop were therefore related to methods, tools, applications, and perspectives on structural bioinformatics, proteomics and integrative systems biology. These issues are very relevant for several research communities which were invited to join forces and create synergies for an interdisciplinary effort aimed at developing new tools at the interfaces of these disciplines.

The programme included four keynote and three special guest lectures, as well as 15 oral communications. The keynote talks were given by Raffaele Calogero, University of Torino, Italy, Wolfgang Marwan, Otto-von-Guericke Universität, Magdeburg, Germany, Torsten Schwede, University of Basel, Switzerland, and the Nobel Prize winner in Chemistry in 2009, for her studies of the structure and function of the ribosome, Ada Yonath, Weizmann Institute of Science, Rehovot, Israel.

Raffaele Calogero gave a talk on “Exosome transcriptome analysis to provide novel tools for CLL patient stratification”, where an exhaustive analysis of the transcriptome of exosomes circulating in the peripheral blood of leukemia (CLL/MBL) patients was presented. The data presented, still unpublished, suggest that the exosome transcriptome of CLL and MBL patients carries an important amount of non-coding RNAs, which might be instrumental for CLL patients’ stratification.

“Biomodel engineering with Petri nets” was the title of the talk given by Wolfgang Marwan. With Petri nets as formal language for biomodel engineering, he described the general concept of a modular modelling approach that considers the functional coupling of modules representing components of the genome, the transcriptome, and the proteome in the form of an executable model. This approach has been recently published in [5].

Torsten Schwede talked about “Modeling protein structures and complexes using evolutionary information”. Structure modelling has traditionally been focused on predicting the 3D structure of a single protein chain, although the majority of proteins in a living cell exist as part of complexes and quaternary structure assemblies. However, ligand binding sites and enzyme active sites are often located between protein chains, and mutations in protein-protein interfaces are often related to diseases. In his talk, Schwede presented an approach for comparative protein quaternary structure modelling using an evolutionary interaction fingerprint profile. A useful reference for this approach can be found in [6].

Finally, “Crystallizing macromolecular complexes: the ribosome” was the title of Ada Yonath’s talk. The Nobel Laureate’s talk, which was given in a joint session with the “2014: Crystal (cl)Year” Conference, was focused on the long process that was necessary to solve the three dimensional structure of the ribosome, starting from the discovery of the crystallization conditions to the final model building and refinement. Yonath’s talk captured the attention of all those present for its clarity, thrilling young researchers and, in general, all participants.

Two full day hands-on tutorials were given in parallel by researchers of the University of Torino. Giovanna Di Nardo talked about “Protein engineering and drug design based on 3D protein models”, and Raffaele Calogero and Francesca Cordero gave a tutorial on “Next generation data analysis using OneChannelGUI”.

## Selection of best papers

Ten full papers were submitted for publication in this Supplement after the workshop. An Editorial Board was formed by paying attention that topics of submitted manuscripts were properly covered. It included the following Associated Editors:

- Francesca Cordero, University of Torino, Italy
- Angelo Facchiano, CNR - Istituto di Scienze dell'Alimentazione, Avellino, Italy
- Helena Galhardas, University of Lisbon and INESC-ID, Lisbon, Portugal
- Stefano Lonardi, University of California Riverside, USA
- Lars Gustav Malmström, University of Zurich, Switzerland
- Stefano Pascarella, Università “La Sapienza”, Roma, Italy
- Paolo Romano, IRCCS AOU San Martino IST, Genova, Italy
- Torsten Schwede, Swiss Institute of Bioinformatics, Basel & University of Basel, Switzerland

Each Associate Editor managed the reviewing process for one or two papers, according to his/her expertise. At least two, but often three, referees were selected for each submission, and overall 20 referees were involved. A two-step peer review procedure was adopted: some of the authors were invited to submit a revised version of their paper, according to the referees’ comments and the associated editor recommendation, before the final decision was taken. Associated Editors made the final recommendation for each paper and, at the end of this process, five papers were accepted and included in this Supplement.

## A short presentation of selected papers

Not all workshop topics were addressed by submissions, but the papers that have been selected for this publication reflect well the overall complexity of the theme, ranging from visualization of molecular interaction networks to molecular dynamics applications, and from the automated analysis of mass spectra to the integration of multi-omic information.

Ceol A et al. are the authors of “Genome and network visualization facilitates the analyses of the effects of drugs and mutations on protein-protein and drug-protein networks” [7]. In this work, where structural, drug binding and network databases are combined to identify key residues for protein-protein interactions, a new plugin for the Integrated Genome Browser (IGB) is presented. The plugin applies recent visualization techniques to the analysis of genomic data by means of network and structural approaches. The assumption of the authors is that three dimensional representations of interactomes may support the identification of disease-relevant protein interaction sites, which can be identified as drug targets, thanks to the advances in molecular interaction and structure visualization tools, which simplify the mapping of mutated residues to molecular interaction interfaces. Many examples of this approach are presented in the paper.

The manuscript “A novel molecular dynamics approach to evaluate the effect of phosphorylation on multimeric protein interface: the αB-Crystallin case study” [8] by Chiappori F et al. describes a new method that, although presented here for the special case of two candidate proteins (aGP and TBK1) aimed at building a model for the evaluation of effects in αB-Crystallin, could likely be generalized and become of use to a large community of researchers. The method is based on the execution of short molecular dynamics simulations of different phosphorylation states followed by an analysis based on predefined properties, such as solvent accessible surface and the number of hydrogen bonds, to be calculated from the molecular dynamics trajectories.

In their work “Fast, accurate, and lightweight analysis of BS-treated reads with ERNE 2” [9], Prezza N et al. describe the software tool ERNE 2 for mapping bisulfite treated reads and making methylation calls, a data analysis field which is computationally expensive. Reducing required computing time and memory is therefore an important goal. A relevant characteristic of the algorithm behind the tool is the implementation of a compact and efficient database hash data structure. Also important is the ability of the tool to deal with targeted bisulfite sequencing, which is achieved by supplying coordinates of the target regions so that methylation calls can be reported for those regions only. The performance of ERNE 2 is compared to the well-known aligner and methylation caller for bisulfite-seq applications Bismark [10], both on an actual and on a simulated dataset, by achieving brilliant scores both in term of speed and results.

The paper “Geena 2, improved automated analysis of MALDI/TOF mass spectra” [11] by Romano P et al. presents a web application for the automation of a pipeline concerning MALDI/TOF mass spectra data pre-processing. In this context, many steps, such as joining isotopic abundances for the same peptide, normalizing signals against internal standards, and removing background noise, are required. Moreover, when many spectra from the same sample (replicate spectra) and from different patients must be analyzed, e.g., for biomarker discovery or classification purposes, an average spectrum for each sample and an alignment of spectra from different samples must also be created. Geena 2 was implemented to carry out these preliminary analysis for MALDI/TOF spectra and it is accessible through three simple and intuitive web interfaces. Its use in published experimental works is also reported in the paper.

The manuscript “Weighted integration of multi-omic layers of conditions in genome-scale models” [12] by Angione C et al. presents a novel framework for integration of multiple omic data types in the context of genome-scale metabolic models. In the paper, the framework is used to integrate transcriptomic data with predicted metabolic flux data in over 2,000 conditions for E. coli. Such integration allowed to identify metabolic reactions able to differentiate groups of similar conditions and, thus, to assess observed or predicted differences in cellular metabolic states.

## Abbreviations

BS: Bisulfite
CLL: chronic lymphocytic leukemia
ERNE: Extended Randomized Numerical alignEr
ICT: Information and Communication Technologies
IGB: Integrated Genome Browser
MALDI/TOF: Matrix Assisted Laser Desorption Ionization Time-of-Flight
MBL: Monoclonal B-cell lymphocytosis
NETTAB: Network Tools and Applications in Biology
XML: Extensible Markup Language
