# Erratum to: ‘NETTAB 2014: From high-throughput structural bioinformatics to integrative systems biology’

**DOI:** 10.1186/s12859-016-1050-5

**Published:** 2016-07-29

**Authors:** Paolo Romano, Francesca Cordero

**Affiliations:** 1 Bioinformatics, IRCCS AOU San Martino – IST, c/o Centro Biotecnologie Avanzate (CBA), Largo Rosanna Benzi 10, I-16132 Genoa, Italy; 2 Department of Computer Science, University of Torino, Torino, I-10149 Italy

Unfortunately, the original version of this article [1] contained a few errors. The editorial department of *BMC Bioinformatics* would like to apologize and inform its readers of the following revisions.

The first sentence of the last paragraph “The manuscript “Weighted integration of multi-omic layers of conditions in genome-scale models” [12]” should be “The manuscript “Multiplex methods provide effective integration of multi-omic data in genome-scale models” [12]”.

In the reference list, the title of reference [12] “Weighted integration of multi-omic layers of conditions in genome-scale models” should be “Multiplex methods provide effective integration of multi-omic data in genome-scale models”.

Reference 7 has 2016;17 Suppl 3:S2 as its year, volume and supplement number and this should be 2016;17(Suppl 4):54 instead.

Reference 8 has 2016;17 Suppl 3:S3 as its year, volume and supplement number and this should be 2016;17(Suppl 4):57 instead.

Reference 9 has 2016;17 Suppl 3:S4 as its year, volume and supplement number and this should be 2016;17(Suppl 4):69 instead.

Reference 11 has 2016;17 Suppl 3:S5 as its year, volume and supplement number and this should be 2016;17(Suppl 4):61 instead.

Reference 12 has 2016;17 Suppl 3:S6 as its year, volume and supplement number and this should be 2016;17(Suppl 4):83 instead.
